# Pulmonary hypertension and vascular remodeling in mice exposed to crystalline silica

**DOI:** 10.1186/s12931-016-0478-5

**Published:** 2016-11-28

**Authors:** Igor N. Zelko, Jianxin Zhu, Jeffrey D. Ritzenthaler, Jesse Roman

**Affiliations:** 1 Department of Medicine, Division of Pulmonary, Critical Care, and Sleep Medicine, University of Louisville, Louisville, KY 40202 USA; 2 Department of Biochemisry and Molecular Genetics, University of Louisville, Louisville, KY 40202 USA; 3 Robley Rex VA Medical Center, Louisville, KY 40202 USA

**Keywords:** Silicosis, Pulmonary hypertension, Vascular remodeling, Animal model

## Abstract

**Background:**

Occupational and environmental exposure to crystalline silica may lead to the development of silicosis, which is characterized by inflammation and progressive fibrosis. A substantial number of patients diagnosed with silicosis develop pulmonary hypertension. Pulmonary hypertension associated with silicosis and with related restrictive lung diseases significantly reduces survival in affected subjects. An animal model of silicosis has been described previously however, the magnitude of vascular remodeling and hemodynamic effects of inhaled silica are largely unknown. Considering the importance of such information, this study investigated whether mice exposed to silica develop pulmonary hypertension and vascular remodeling.

**Methods:**

C57BL6 mice were intratracheally injected with either saline or crystalline silica at doses 0.2 g/kg, 0.3 g/kg and 0.4 g/kg and then studied at day 28 post-exposure. Pulmonary hypertension was characterized by changes in right ventricular systolic pressure and lung histopathology.

**Results:**

Mice exposed to saline showed normal lung histology and hemodynamic parameters while mice exposed to silica showed increased right ventricular systolic pressure and marked lung pathology characterized by a granulomatous inflammatory reaction and increased collagen deposition. Silica-exposed mice also showed signs of vascular remodeling with pulmonary artery muscularization, vascular occlusion, and medial thickening. The expression of pro-inflammatory genes such as TNF-α and MCP-1 was significantly upregulated as well as the expression of the pro-remodeling genes collagen type I, fibronectin and the metalloproteinases MMP-2 and TIMP-1. On the other hand, the expression of several vasculature specific genes involved in the regulation of endothelial function was significantly attenuated.

**Conclusions:**

We characterized a new animal model of pulmonary hypertension secondary to pulmonary fibrosis induced by crystalline silica. Our data suggest that silica promotes the damage of the pulmonary vasculature through mechanisms that might involve endothelial dysfunction, inflammation, and vascular remodeling.

## Background

Exposure to silica may occur in a variety of working and living environments since crystalline silica is one of the most abundant minerals on earth. For example, occupational expose to silica occurs during mining, stone cutting, tunneling and quarrying [1]. Environmental exposure to silica may occur during sand storms, during inhalation of very fine particles of windblown soil, and following volcanic eruptions. Chronic inhalation of crystalline silica promotes the development of several diseases such as silicosis, chronic obstructive pulmonary diseases (COPD), and lung cancer [2, 3]. Silicosis is a fibrotic pneumoconiosis characterized by nonneoplastic granulomatous and fibrotic changes in the lung. Silica-exposed patients remain asymptomatic for decades when eventually diagnosed by the presence of fine nodular opacities in the lung by chest X-ray or CT-scan [4]. Depending of dose and time of exposure, silica may produce acute or various forms of chronic silicosis [5].

In general, two major stages can be defined during silicosis progression. First, an inflammatory stage characterized by the release of inflammatory mediators such as IL-1β, IL-6, TNF-α that can continue to be released into the second fibrotic stage. The second state is a fibrotic stage characterized by excess deposition of extracellular matrix proteins such as collagen and fibronectin [6, 7]. Although the exact mechanisms responsible for these changes remain unclear, it is well established that inhaled silica particles are engulfed by macrophages, which leads to cell activation and death followed by the release of intracellular silica that is then taken up by other macrophages. This recurring cycle of cell death and macrophage activation produces the influx of inflammatory cells and the production of cytokines and reactive oxygen and nitrogen species [8]. These inflammatory mediators are able to enter the pulmonary and systemic circulations where they can produce vascular injury. Moreover, ultra-fine silica particles may cross the pulmonary epithelium into the vascular bed and directly affect the integrity of the vascular endothelium [9, 10]. Interestingly, cardiovascular diseases are among the leading causes of death in patients with silicosis [11].

The recurring injury to the pulmonary vasculature may lead to the development of pulmonary hypertension. Pulmonary hypertension results from a proliferative vasculopathy of the small pulmonary arteries and arterioles of the lung best characterized by vasoconstriction, cellular hyperplasia, fibrosis, and thrombosis. These constricted or blocked arteries lead to increased pressure in the vessels and in the right ventricle of the heart. If left untreated, the right ventricular chamber hypertrophies leading to premature right heart failure. In the United States, about 200,000 hospitalizations occur annually due to pulmonary hypertension as primary or secondary diagnosis. About 15,000 deaths per year are ascribed to pulmonary hypertension, although this is likely a low estimate [12]. The contribution of silicosis to these statistics is likely small, but it has been shown that pulmonary hypertension in patients with silicosis is linked with poorer prognosis [13]. Similarly, COPD and diffuse parenchymal lung diseases, including idiopathic lung fibrosis and sarcoidosis, are associated with a high incidence of pulmonary hypertension [14, 15]. Patients with combined interstitial lung disease and pulmonary hypertension have significantly lower survival rate and quality of life.

Because of its clinical relevance, elucidating the mechanisms by which silicosis may lead to pulmonary hypertension is considered important. Animal models of silica exposure exist and, as observed in humans, silica induces granulomatous changes in the lungs of these animals, resulting from the loose aggregation of activated foamy histiocytes and lymphocytes or well organized nodular structures consisting of epitheloid macrophages and multinucleated giant cells [16, 17]. However, there are currently no well-described in vivo models of silica exposure that demonstrate increased risk of pulmonary vascular remodeling and pulmonary hypertension. The objective of this study was to determine whether silica exposure leads to increased right ventricular systolic pressure (RVSP) and vascular remodeling in pulmonary arteries. We now provide evidence that RVSP and vascular abnormalities are markedly increased in silica-exposed mice compared to control mice.

## Methods

### Experimental animals and animal care

The research protocol was approved by the Institutional Animal Care and Use Committee of the University of Louisville, and the care and handling of the animals were in accordance with National Institutes of Health guidelines. C57BL6 mice were obtained from Jackson Laboratory (Bar Harbor, ME).

### Animal model

Adult male C57BL6 mice (10 weeks of age) were separated into 5 experimental groups with 5 animals per group. Animals were anesthetized and placed in the supine position. Using sterile technique, the trachea was exposed via midline neck incision followed by instillation of silica or saline. Crystalline silica was sterilized at 200 °C for 2 h to inactivate endotoxin contamination. Silica suspension in sterile 0.9% NaCl was prepared by vigorous vortexing immediately prior to intratracheal administration. Using a 27-gauge needle attached to a microliter syringe, 0.2 g/kg, 0.3 g/kg and 0.4 g/kg of crystalline silica suspension or the equivalent volume of saline was instilled into the trachea. The fifth group of mice was instilled with 3.5 U/kg of bleomycin (APP Pharmaceuticals, Schaumburg, IL). The incision was then closed using surgical clips, and animals were allowed to recover. Twenty-eight days after intratracheal instillation of silica, RSVP parameters were measured and lung and heart tissues were harvested for morphological, biochemical, and histochemical analyses. Bleomycin-injected mice (3.5 U/kg) were used as positive control and were analyzed 21 days later. Tissue was immediately processed or quick-frozen in liquid nitrogen.

### Reagents

Primers and probes for real-time PCR were obtained from Integrated DNA and ThermoFisher Scientific. All other chemicals and enzymes were from Sigma Chemical Co. (St. Louis, MO), or Invitrogen (Carlsbad, CA). Crystalline silica was a gift from US Silica (Min-U-Sil-5, US Silica, Frederick, MD).

### Hemodynamic measurements

RVSP was determined with a 1 F pressure transducer catheter (Millar Instruments) and LabChart 8 software (AD Instruments). Briefly, the 1 F pressure transducer was inserted through the right external jugular vein of anesthetized mice (100 mg ketamine/5 mg xylazine/kg of body weight, i.p.). Mice were placed on thermal plates to keep body temperature constant at 37 °C. Then, a pressure catheter was threaded into the right ventricle and RVSP was recorded using PowerLab 4/35 (AD Instruments) and analyzed using LabChart 8 software.

### Lung and heart histology

Heart and lungs were flushed with PBS and inflated and fixed with 10% formalin overnight, then embedded in paraffin, sectioned at a thickness of 5 μm, and stained with Mason’s trichrome to visualize lung morphology, fibrosis and vascular remodeling. Images were captured by a high-resolution digital camera connected to a light microscope using 4× and 40× magnification lenses. The evaluation and image analysis procedures were performed using ImageJ software.

### Immunohistochemical staining of mouse lungs for smooth muscle, von Willebrand Factor (vWF), LY-6B and CD107b

Longitudinal sections (5 μm) of left lung lobe were hydrated and antigen retrieval was first performed by incubating with 0.1% pronase for 5 min at 37 °C and then heating the slides in 10 mM sodium citrate (pH 6.0) plus 0.05% Tween 20 at 98 °C for 10 min. Sections were stained with anti-smooth muscle actin-alpha antibodies clone 1A4 (Sigma) at concentration 23 ng/μl, anti-vWF antibodies H-300 (Sigma), anti-LY-6B and anti CD-107b antibodies (Bio-Rad) at concentration 10 ng/μl. After washing, the slides were incubated with secondary antibodies labeled with either AlexaFluor 488 or AlexaFluor 594. To determine the specificity of staining, lung sections were incubated with control, non-immune IgG. Slides were analyzed with fluorescent microscopy. Images were processed using ImageJ (National Institutes of Health, Bethesda, MA). Pulmonary arteries were defined as vessels that accompanied airways (veins are interlobular). To measure percent of area stained with specific marker of neutrophils and macrophages we used ImageJ software. For silica and bleomycin treated lung at least four different areas showing pulmonary arteries and silicotic granulomas or fibrotic lesions were selected. The stained areas were selected by thresholding and then calculated using particles analysis extension of ImageJ. The same parameters for thresholding and for calculation of particles were applied to all images.

### Right ventricular hypertrophy

After hemodynamic measurements, the hearts were removed and right and left ventricles and septum were separated. The ratio of the right ventricular weight to the sum of left ventricular and septal weight (RV/[LV + S]) served as a measure for right ventricular hypertrophy.

### Granuloma area calculation

In sections stained with Mason’s trichrome, the total area of lung section and granuloma area were determined using ImageJ software. Three to four images of each lung were taken at 40× magnification to cover entire lung lobe. Granuloma area percentage was calculated by dividing granuloma area by total lung area and multiplying by 100.

### Pulmonary vessels morphometry

To assess muscularization of pulmonary vessels, all blood vessels ranging from 10–100 μm in diameter were counted in at least four fields at 40× magnification. The counted vessels were categorized as fully muscularized (95–100% of medial layer covered by anti-αSMA staining), partially muscularized (1–95% of medial layer is covered by anti-αSMA staining), or nonmuscularized vessels. The percentage of pulmonary vessels in each category was calculated by dividing the number of vessels in the category by the total number of counted vessels in the same field.

### Morphometric analysis

The diameter and wall thickness of arteries were measured using ImageJ software, after the number of pixels were calibrated according to the scale bars for each magnification. The values of medial wall thickness were calculated as outer diameter minus inner diameter divided by 2. At least four vessels were counted for each mouse lung. The analysis and measurement of α-SMA staining was evaluated by an investigator blinded to treatment groups.

### Quantitative RT-PCR

Total RNA was prepared from the superior lobe of right lung using RNAqueous-Micro Kit (Applied Biosystems, Foster City, CA). The synthesis of single stranded DNA from RNA was performed using SuperScript First-Strand Synthesis System for RT-PCR and random hexamers (Invitrogen, Carlsbad, CA), according to the protocol provided by manufacturer. To quantitate the abundance of gene-specific mRNAs, quantitative PCR was undertaken using the StepOnePlus Real-Time PCR Detection System (Applied Biosystems) and an SYBR® Green Master Mix. The PCR cycles were 95 °C for 3 min, then 40 cycles of 95 °C for 15 s, 60 °C for 1 min. The mouse fibronectin primers were forward (5′- GAC TGT ACT TGT CTA GGC GAA G -3′) and reverse (5′-GTT TCC TCG GTT GTC CTT CT-3′), mouse PECAM-1 primers were forward (5′-AGA GAC GGT CTT GTC GCA GT-3′) and reverse (5′-TAC TGG GCT TCG AGA GCA TT-3′), mouse Endothelin 1 primers were forward (5′- TCT GCA CTC CAT TCT CAG C-3′) and reverse (5′- CGT GAT CTT CTC TCT GCT GTT C-3′), mouse Platelet Factor 4 (PF4) primers were forward (5′- ACC ATC TCC TCT GGG ATC CAT-3′) and reverse (5′-CCA TTC TTC AGG GTG GCT ATG AG-3′), mouse Nestin primers were forward (5′-GGA AAG CCA AGA GAA GCC T-3′) and reverse (5′-CAC CTC AAG ATG TCC CTT AGT C-3′). PCR assays were run in triplicate, and gene expression was normalized to β-Actin mRNA levels. Primers for β-Actin were forward (5′- ACA GCT TCT TTG CAG CTC CT-3′) and reverse (5′-CCA TCA CAC CCT GGT GCC TA-3′). Analysis of Col1a1, Timp1, Ctgf, Tnf and Mmp2 were performed using custom gene expression assays with FAM labeled probe obtained from Applied Biosystems. The mRNA levels for these genes were normalized for β-Actin mRNA levels that were detected with custom gene expression assay with VIC labeled probe.

### Data analysis

Values were expressed as means ± SEM. Comparisons between multiple independent groups were made by using One-way ANOVA followed by post hoc analysis with the Holm-Sidak test. Data of two groups were compared with unpaired t-test. A *p*-value of <0.05 indicated statistically significant differences.

## Results

### Silica exposure causes weight loss

**Fig. 1:**
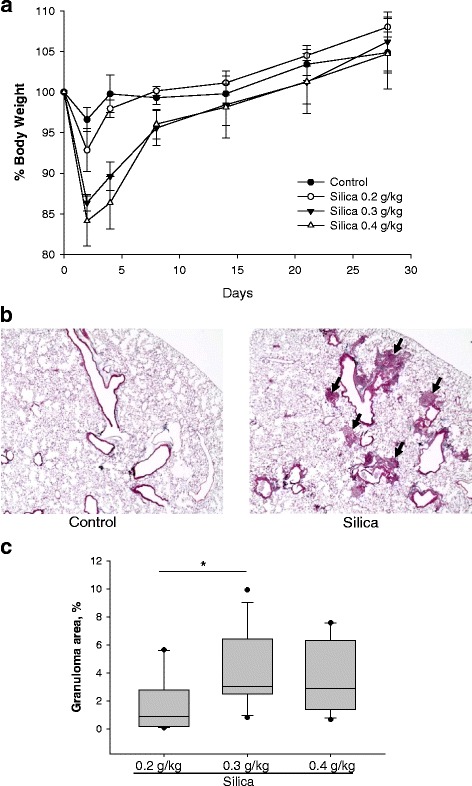
Weight loss and silicotic nodules in mice treated with crystalline silica. C57BL6 male mice were intratracheally injected with different amount of crystalline silica suspension. Mice were sacrificed 28 days later. **a**, Silica-induced body weight changes in mice were measured at 2, 4, 7, 14, 21 and 28 days following silica administration. Mice receiving higher doses of silica showed more weight loss. Results are expressed as the mean of five mice +/− SD. **b**, Morphologic evidence of silica-induced lung injury. Representative images of lung from control and silica-treated mice. Arrows indicate granulomatous lesions. **c**, Quantitative analysis of granulomatosis in lung of silica-treated mice. *p < 0.05 when compared between treatments, One Way ANOVA with Holm-Sidak post test

Instillation of increasing amounts of crystalline silica into the trachea of animals led to the progressive loss of body weight at day 2 and day 4 after the start of the treatment (Fig. 1a). With a silica dose of 0.4 g/kg, mice lost up to 16% of their body weight, but regained close to the weight of the control group by day 14. This quick recovery of body weight is in contrast with our observations after bleomycin treatment where weight loss was more severe and maximum body weight loss was observed around day 10 (data not shown). Bleomycin-treated mice did not regain their body weight to the level of control mice even at day 21. Similarly to the weight loss, survival of mice after treatment was affected only in the bleomycin group (2 mice out of total 5 mice died (40% mortality).

### Silica-induced lung fibrosis

To confirm the effects of silica instillation on the pathogenesis of lung inflammation and fibrosis, the lung tissues of mice were observed using light microscopy to monitor pathological changes. No obvious abnormalities were evaluated in the lungs of mice that received saline at days 28 (Fig. 1b, Left Image). Conversely, cellular nodules, fibrotic bands, and marked collagen deposition determined by trichrome staining were observed in the silica-treated groups. Quantitative analysis of the area of the fibrotic nodules indicated significant and dose-dependent increase of lesion area in the lung of silica-treated mice (Fig. 1c). These data correlated with loss of body weight depicted in Fig. 1a.

### Crystalline silica increases right ventricle (RV) systolic pressure

**Fig. 2:**
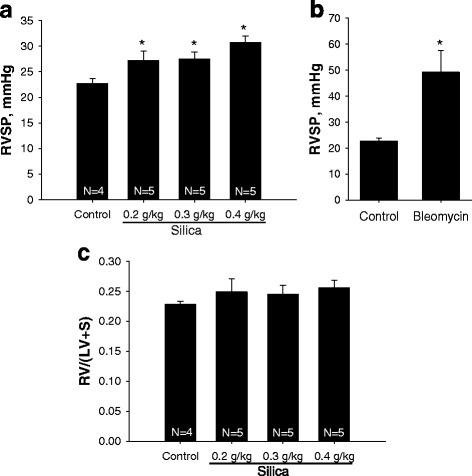
Elevation of right ventricular systolic pressure in mice treated with silica. Mice treated with different concentration of silica for 28 days (**a**) or bleomycin for 21 days (**b**) were analyzed for right ventricle systolic pressure (RVSP) using 1 F pressure-transducer catheter (Millar). **c**, Ratio of right ventricle (RV) to left ventricle (LV) plus septum (S) was determined by dissecting RV from LV and S. The results are shown as mean ± SEM, *p < 0.05 when compared to control lung, One Way ANOVA with Holm-Sidak post test

To evaluate the dose-dependent effects of silica on right ventricle systolic pressure (RVSP), mice were anesthetized and RV systolic pressure was measured using pressure catheter. Significant changes in RVSP were observed in silica-treated mice (from 22.65 ± 1.01 mmHg in control to 30.75 ± 1.28 mmHg; *p* < 0.05) in mice treated with silica at dose 0.4 g/kg (see Fig. 2a). Treatment of mice with bleomycin increased RVSP to 49.21 ± 8.276 mmHg (Fig. 2b). However, no changes in RV hypertrophy measured as ratio of RV/(S + LV) were observed (Fig. 2c). These data indicate that silica exposure induced pulmonary hypertension, but the latter was either not severe enough to cause RV hypertrophy or more time was required to observe these effects.

### Morphological changes in pulmonary arteries of silica-treated mice

**Fig. 3:**
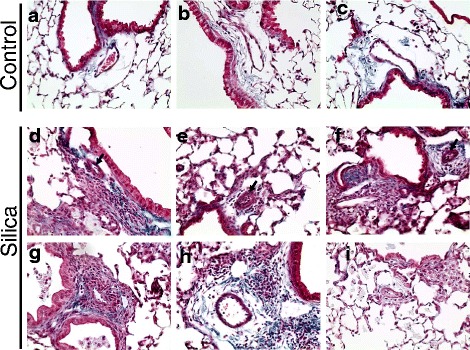
Histological analysis of vascular abnormalities in lung from silica-treated mice. Mouse lung were stained with Mason’s trichrome and imaged using 400× magnification. Panel **a**-**c**: representative images of pulmonary arteries from control mice. Panel **d**-**i**: images of pulmonary arteries with different vascular abnormalities. Vascular wall thickening (**d**-**f**); inflammatory cells and collagen deposition around pulmonary arteries (**g**-**h**); endothelial proliferation (**i**)

Next, we analyzed the effect of silica on pulmonary vascular remodeling. Control mice showed normal pulmonary artery structures (Fig. 3a-c). Mice exposed to crystalline silica through intratracheal instillation developed marked vascular remodeling. The most obvious vascular abnormalities observed were detected near inflammatory granules and fibrotic lesions. The representative images presented in Fig. 3d-f indicated that pulmonary arteries showed vascular wall thickening (see arrows). In addition, vessels located in close proximity to inflammatory nodules in some cases became completely surrounded by inflammatory cells (Fig. 3g) and/or surrounded by collagen fibrils (Fig. 3h). Intimal hypertrophy was observed in some pulmonary vessels (Fig. 3i). These histological observations indicate that pulmonary vascular walls undergo significant remodeling in the lungs of silica-treated mice.

### Induction of inflammatory and extracellular matrix genes

**Fig. 4:**
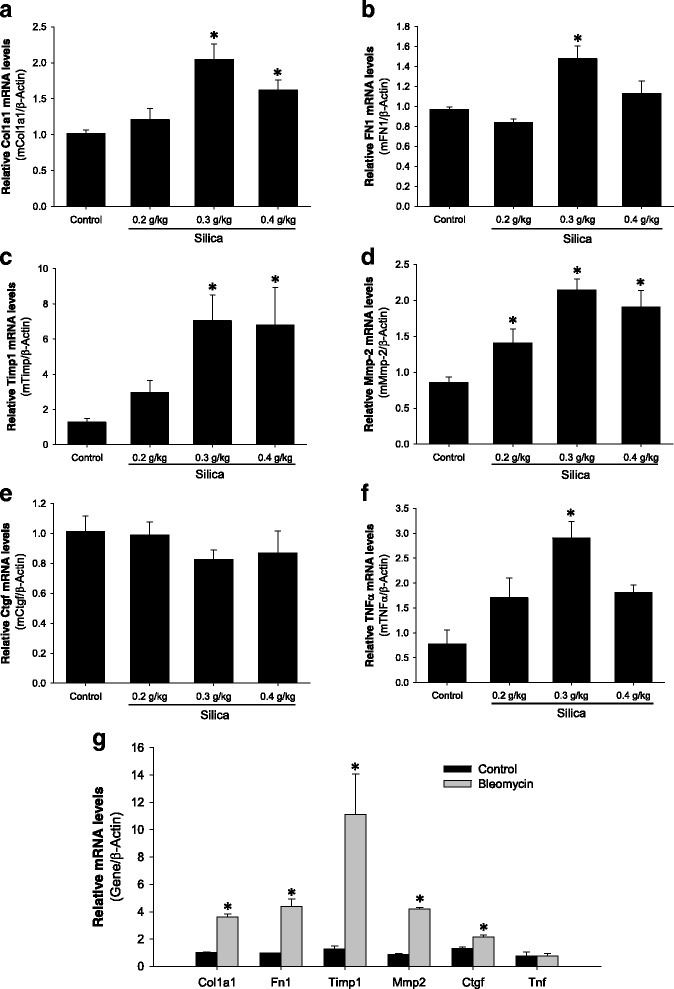
Silica exposure increases pro-fibrotic gene expression. Analysis of gene expression in the lung of silica treated mice (panels **a**-**f**). Whole lung mRNA levels for collagen type I, alpha 1 (Col1a1), fibronectin 1 (Fn1), tissue inhibitor of metalloproteinases 1 (Timp1), matrix metalloproteinase 2 (Mmp-2), connective tissue growth factor (Ctgf) and tumor necrosis factor alpha (TNF-α) were determined using real-time PCR and normalized to beta-acting expression (β-Actin). **g**, Changes in gene expression in the lung of bleomycin treated mice at 21 days. The results are shown as mean ± SEM, *p < 0.05 when compared to control lung, One Way ANOVA with Holm-Sidak post test

Quantitative real-time PCR was performed to measure the levels of collagen type I (Col1a1), fibronectin (Fn1), TIMP-1, MMP-2, CTGF and TNF-α mRNAs in the right lung. The mRNA expression of collagen type I and fibronectin was markedly elevated the lungs of animals exposed to silica at a dose of 0.3 g/kg and 0.4 g/kg, indicating the induction of pro-fibrotic genes in silica-treated lungs (Fig. 4a-b). Even greater increases were detected for mRNA levels of TIMP-1 (7 fold) and of MMP-2; (2.2 fold), over control mice (Fig. 4c-d). The expression of pro-inflammatory mediators TNF-α and MCP-1 was also increased from 3- to 5-fold compared to control mice (Fig. 4f and data not shown). Increases in the mRNA expression of these genes were also detected in the bleomycin model (Fig. 4g). On the other hand, no induction of CTGF mRNA expression was observed in silica-treated mice in contrast to bleomycin-treated lungs (Fig. 4e and g). Interestingly, while expression of Col1a1, Fn1, Timp1 and Mmp2 genes was induced to a higher extent in bleomycin-treated lungs compared to silica-treated lungs, the expression of the TNF-α gene was unchanged in the bleomycin group (Fig. 4g). These data indicate that silica induces significant changes in expression of pro-fibrotic and pro-inflammatory genes in the mouse lung, but there are some differences when compared to the bleomycin model.

### Silica promotes pathological signs of pulmonary vascular remodeling and medial thickening

**Fig. 5:**
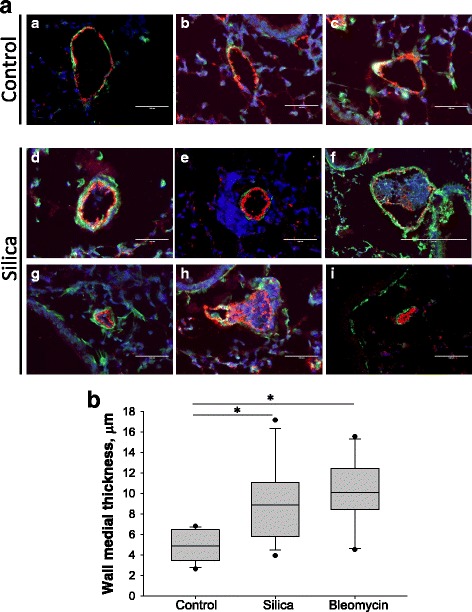
Abnormal endothelial and smooth muscle remodeling in the pulmonary vasculature of silica-treated mice. **a**, Lung sections (5 μm thick) were stained with antibodies specific for endothelium-specific von Willebrand Factor vWF (*red*) and α-smooth muscle actin (*green*). Nuclei were stained using DAPI (*blue*). Images were taken using 40× objective. Panel **a**-**c**: representative images of pulmonary arteries from control mice. Panel **d**-**i**: images of pulmonary arteries with different vascular abnormalities: increased accumulation of inflammatory cells around vessel (**e**), intimal obliteration and/or luminal occlusion by vWF-positive cells (**f** and **h**), neointimal proliferation (**g** and **i**). Bar = 100 μm. **b**, Quantitative analysis of medial wall thickness in pulmonary arteries. *p < 0.05 when compared to control lung, One Way ANOVA with Holm-Sidak post test

In order to identify vascular abnormalities in silica-treated mice, lung sections were stained with antibodies specific for α-smooth muscle actin (for smooth muscle cells) and von Willebrand Factor (for endothelial cells). While the vasculature from control mice showed normal pulmonary artery architecture (Fig. 5a images a-c), vascular remodeling was apparent in the lungs of mice exposed to crystalline silica (Fig. 5a images d-i). Interestingly, a complex stalk-like lesion was detected within a blood vessel lumen (Fig. 5a image h). It was formed just distal to a dichotomous branching point. The body of the lesion appeared to be a disorganized hyperchromatic accumulation of cells that were covered by von Willebrand factor–positive endothelial cells. The stalk-like structure appeared to arise from the arterial wall and extend downstream into the lumen of the vessel (Fig. 5a image f). Moreover, quantitative analysis of medial wall thickness indicated significant increase in thickness of smooth muscle layer from 4.85 ± 0.42 μm in control lung to 9.14 ± 0.93 μm (*p* < 0.05) in silica treated lung and to 10.21 ± 1.04 μm (*p* < 0.05) in bleomycin treated lung (Fig. 5b).

### Muscularization of pulmonary arteries in response to silica

**Fig. 6:**
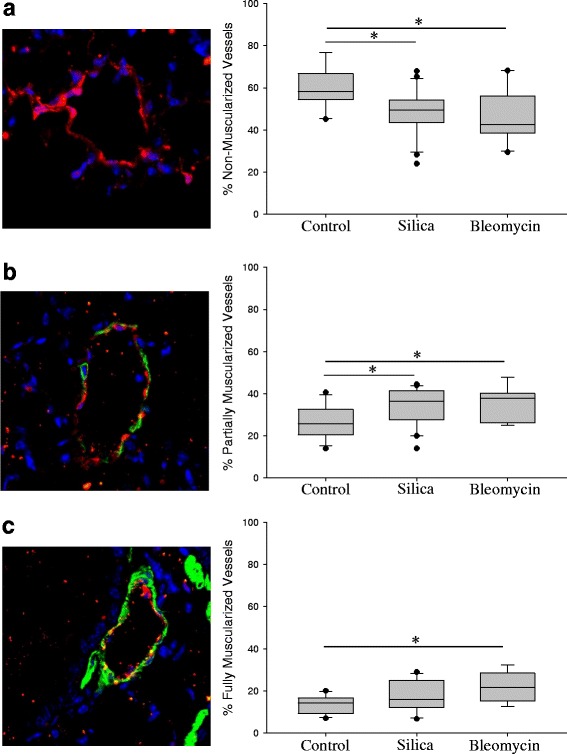
Muscularization of pulmonary arteries in silica- and bleomycin-treated mice. Lung sections were stained with vWF (*red*) and alpha-smooth muscle actin (*green*) specific antibodies. Nuclei were stained using DAPI (*blue*). Representative images of non-muscularized (**a**), partially muscularized (**b**) and fully-muscularized (**c**) arteries showed on left side. Quantitative analysis of pulmonary arteries remodeling presented on right side. Significant increase in partially-muscularized and fully muscularized arteries was detected in silica (0.4 g/kg) and bleomycin-treated lungs

Treatment of mice with both silica and bleomycin resulted in a decrease in the number of non-muscularized pulmonary vessels (Fig. 6a). These changes were associated with significant increases in partially muscularized and fully muscularized vessels. Treatment with 0.4 g/kg silica increased the percent of partially muscularized vessels from 26.85 ± 2.03 to 33.51 ± 1.92 (*p* < 0.05). At the same time, the percentage of fully muscularized vessels increased from 13.63 ± 1.18 in control mice to 17.67 ± 1.69 (not significant) in silica-treated mice and to 22.30 ± 2.36 (*p* < 0.05) in bleomycin treated mice (Fig. 6c). Thus, our data indicate that crystalline silica and bleomycin produced comparable changes in pulmonary vessel muscularization.

### Modulation of vascular-specific gene expression in silica-treated mice

**Fig. 7:**
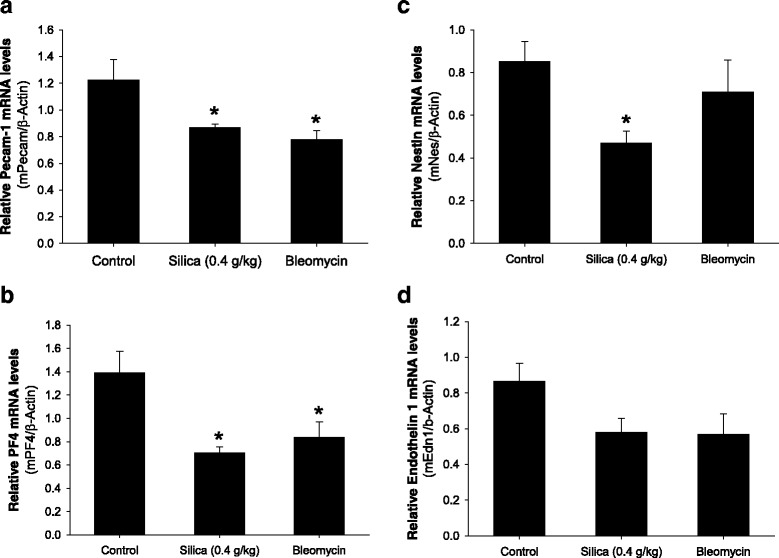
Expression of vascular-specific genes. Analysis of gene expression in the lung of silica- and bleomycin-treated mice (panels **a**-**d**). Expression of genes was analyzed 28 days following silica instillation and 21 days following bleomycin instillation. Whole lung mRNA levels for PECAM-1 (CD31), platelet factor 4 (PF4), nestin and endothelin 1 were determined using real-time PCR and normalized to beta-acting expression (β-Actin). The results are shown as mean ± SEM, *p < 0.05 when compared to control lung, One Way ANOVA with Holm-Sidak post test

Exposure of murine lung to crystalline silica for 4 weeks attenuated the expression of several endothelium- or smooth muscle-specific genes (Fig. 7). Expression of the endothelium-specific gene CD31 was downregulated both in silica- and bleomycin-treated mice (Fig. 7a). A similar pattern of attenuated expression was observed for platelet factor 4 (Fig. 7b). On the other hand, expression of endothelin 1 remained only slightly attenuated without reaching statistically significant levels in both treatment groups (Fig. 7d), while expression of nestin was attenuated only in silica-treated mice (Fig. 7c). These data indicate that crystalline silica attenuated the expression of genes involved in regulation of cardiopulmonary homeostasis quite similar to bleomycin.

### Increased influx of inflammatory cells into lung of mice treated with silica

**Fig. 8:**
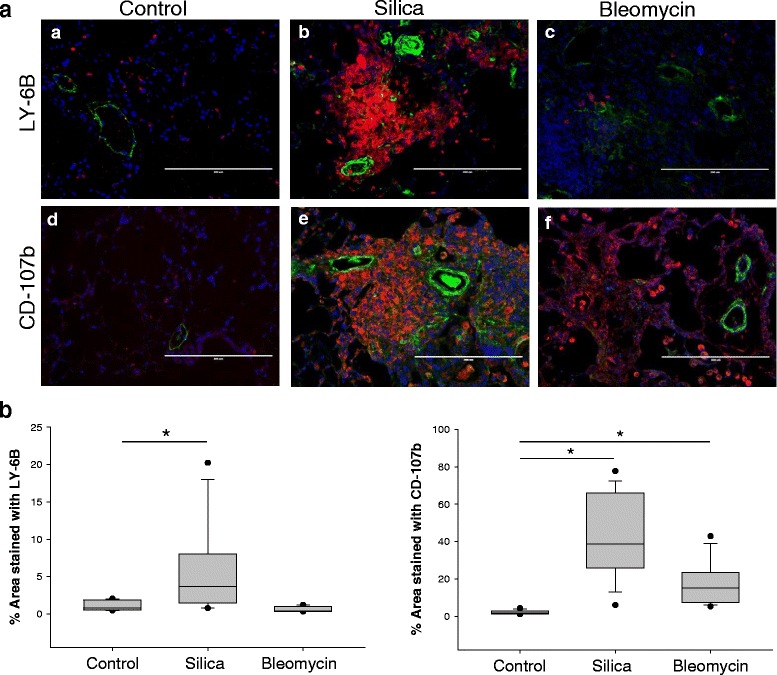
Influx of inflammatory cells in the lung of mice treated with silica and bleomycin. **a**, Lung sections (5 μm thick) were stained with antibodies specific for neutrophil-specific marker LY-6B (*red*), macrophage-specific marker CD107b (red) and α-smooth muscle actin (*green*). Nuclei were stained using DAPI (*blue*). Images were taken using 20× objective. Representative images of mouse lung from control (images **a** and **d**), 0.4 g/kg silica treated (images **b** and **e**) or bleomycin treated (images **c** and **f**) mice. Increased accumulation of inflammatory cells around vessel (images **b** and **e**) found in silica treated lungs. Bar = 200 μm. **b**, Quantitative analysis of area stained with corresponding specific antibodies. *p < 0.05 when compared to control lung, One Way ANOVA with Holm-Sidak post test

Mice treated with silica at concentration of 0.4 g/kg showed significantly higher number of inflammatory cells stained with antibodies specific for LY-6B and CD107b in the lung (Fig. 8). Neutrophils and macrophages were observed mostly in the areas of granuloma location. They were also deposited around vessels adjacent to the lesions (Fig. 8a, images b and e). Surprisingly, we did no observed significant increase in activated peripheral neutrophils in the lung exposed to bleomycin for 21 day (Fig. 8a, image c). In contrast, the number of cells stained with macrophage specific antigen CD107b was increased in bleomycin treated group (Fig. 8a, image e). Quantitative analysis indicates that the CD107b stained area increased from 2.11 ± 0.28% in control lung to 41.31 ± 5.38% (*p* < 0.05) in silica treated lung and to 17.01 ± 3.23% (*p* < 0.05) in bleomycin treated lung (Fig. 8b). Similarly, the area stained with the neutrophil specific marker LY-6B was increased from 1.06 ± 0.17% in control lung to 6.06 ± 1.46% (*p* < 0.05) in silica-treated lung, while no significant changes were observed in bleomycin-treated lungs (Fig. 8b). These data indicate that crystalline silica produced robust inflammatory response that persists even 28 days following initial administration. Attenuated inflammatory staining in bleomycin treated lungs indicated that lung tissues most likely undergo fibrotic remodeling with subsided inflammatory phase.

## Discussion

Despite efforts to prevent occupational exposure to crystalline silica dust, silicosis continues to occur in many developing nations in Asia and South America, and still remains a significant hazard in advanced countries of North America and Europe [18]. Although this disease can be prevented by introducing safer working conditions, equally important is the development of early diagnostic tools and safe and effective therapies for those diagnosed with this devastating disease. Animal models remain an important tool in studying the genesis and the progression pulmonary vascular diseases associated with chronic lung disorders. However, despite strong epidemiologic links between exposure to silica and development of pulmonary hypertension in humans [19], there have been no mouse models described to our knowledge that confirm this association and can be used to elucidate the underlying mechanism(s) responsible. Several previous studies analyzed the effects of asbestos on pulmonary hemodynamic and vascular abnormalities in guinea pigs and rats [20, 21]. The current work suggests that 4 weeks of exposure to crystalline silica is associated with increases in RVSP, vascular remodeling and dysregulation of genes involved in maintenance of vascular homeostasis.

To compare the effects of silica with other injurious agents, we used mice exposed to bleomycin as a model of severe pulmonary hypertension secondary to lung chronic diseases. While many similarities were observed between the two models, we identified several distinctive features that were only seen in silica-exposed mouse lungs. First, the increase in RVSP observed in the silica model was markedly less profound compared to bleomycin model. This difference can be attributed to dosing and exposure differences. How inhaled silica produces its effects on the pulmonary vasculature and hemodynamic is not well understood. It is possible that silica-particles trapped within the lung parenchyma are quickly surrounded by aggregates of thrombocytes and mononuclear leukocytes, raising the likelihood that regional vasoconstriction triggered in response to focally released thromboxane or serotonin could substantially amplify the overall increase in pulmonary vascular resistance [22, 23]. On the other hand, it is possible that reduced cardiac output can significantly obscure pulmonary vascular resistance. Unfortunately, we do not have adequate state-of-the-art equipment to test these possibilities. During the last decade, the proinflammatory cytokines TNF-α and IL-1β have emerged as biomarkers and mediators of oxidative stress and endothelial dysfunction in several cardiovascular diseases [24]. In the present study, we observed that pulmonary arteries from silica-exposed animals showed enhanced TNF-α gene expression despite there being no changes in IL1-β (data not shown). In addition, increased levels of TNF-α can be responsible for the recruitment of more inflammatory cells such as neutrophils and macrophages to the site of injury in silica-treated lungs. Our data indicate that elevated TNF-α gene expression correlates with infiltration of macrophages and neutrophils into silica-induced fibrotic lesions. On the other hand, levels of TNF-α and number of neutrophils did not increase significantly in bleomycin-induced fibrotic lesions. Thus, inhaled silica particles could directly induce endothelial dysfunction by stimulating TNF-α expression and/or by increasing inflammatory cell recruitment in response to elevated secretion of pro-inflammatory cytokines.

We also observed increased expression of MMP-2 and TIMP-1 in lung tissue of silica-exposed mice. MMPs are involved in remodeling of the alveolar architecture near granulomatous lesions but, at the same time, can influence the composition and remodeling of vascular wall. In response to angiogenic stimulus, endothelial-derived MMPs mediate proteolytic degradation of endothelial cell-cell interactions, which promotes a proliferative and migratory phenotype in endothelial cells [25]. In addition, increased MMP-2 and TIMP-1 expression were identified in pulmonary artery smooth muscle cells isolated from idiopathic PAH patients [26]. Importantly, increased gelatinolytic activity was mainly observed in the medial layer, and correlated with increased MMP-2 expression in the pulmonary arteries of monocrotaline-treated animals [27]. This gelatinolytic activity can be attributed to MMP-2 since expression of MMP-9 gene in the pulmonary hypertension model was not observed. The correlation between MMP-2 expression and progression of pulmonary hypertension in the described animal model indicated important roles of this proteinase in different vascular remodeling processes that involves smooth muscle cell proliferation, migration and intimal thickening. The other source of increased MMP-2 and TIMP-1 mRNA levels might be adventitial fibroblasts. Progressing granulomatosis can promote hypoxemia in the lung tissues of silica-treated mice. It has been shown that hypoxia significantly increased MMP-2, TIMP-1, TIMP-2 and α-smooth muscle actin gene expression in adventitial fibroblasts and promoted neointimal hyperplasia [28].

The increase of collagen type I (Col1a1) mRNA levels coinciding with muscularization and thickening of pulmonary vascular wall indicates collagen accumulation in the vasculature. The phenotype switch of pulmonary smooth muscle cell to hypertrophic cells can be regulated by changes in homeostasis of extracellular matrix components like vascular collagen, elastin and fibronectin. Increased expression and deposition of collagen are known to be important determinants of medial thickening during the progression of PAH [29]. Collagen accumulation increases pulmonary arterial stiffening, which translates into pulmonary hypertension progression and eventually, RV dysfunction [30].

We observed significant downregulation in expression of endothelium specific genes such as Pecam 1, also known as CD31, platelet factor 4 (PF4), and nestin. Pecam-1 is expressed on the cell surface of endothelial cells as well as hematopoietic and immune cells including platelets, neutrophils and monocytes. TNF-α and IFN-gamma can reduce the expression of Pecam-1 and transmigration of leukocyte in endothelial cells [31]. Mice deficient in Pecam-1 become hyperresponsive to stimulation with collagen and demonstrate enhanced aggregation and formation of larger thrombi in vitro under physiologic flow conditions [32, 33]. Nestin downregulation in vascular smooth muscle cells represented an early event in vascular disease in experimental type I diabetes [34]. On the other hand, vascular cells expressing nestin were implicated in the development of pulmonary hypertension [35]. Platelet factor 4 (PF4) expression was downregulated in human lung tissue derived from patients diagnosed with pulmonary hypertension secondary to pulmonary fibrosis, but up-regulated in PAH patients [36]. A major physiological role of PF4 is to neutralize heparin-like molecules on the endothelial surface of blood vessels, thereby promoting coagulation and inhibiting angiogenesis. One physiological role of PF4 in endothelial cells is to inhibit endothelial cell growth through multiple signaling mechanisms [37]. Thus, down-regulation of PF4 gene expression in our silica-induced model of pulmonary hypertension might promote angiogenesis and vascular remodeling in affected lungs.

## Conclusions

We demonstrated that exposure of mice to a single intratracheal instillation of crystalline silica causes pulmonary vascular remodeling and pulmonary hypertension in mice, although these changes were less severe than those observed in bleomycin-treated mice. To our knowledge, this is the first study to establish that silica exposure causes vascular abnormalities and mild elevated RVSP in mice. Additionally, we observed significant changes in the expression of genes responsible for inflammatory and fibrotic responses in pulmonary cells as well as genes involved in regulation of vascular function. Together, these observations begin to unveil a mechanistic link between silicosis and pulmonary hypertension. Furthermore, they suggest that the silica-induced murine model of pulmonary hypertension could become a valuable tool to explore the pathogenesis of this disease in humans.

## Abbreviations

COPD: Chronic obstructive pulmonary disease
CTGF: Connective tissue growth factor
IL-6: Interleukin 6
LY-6B: Lymphocyte antigen 6 complex, locus B
MCP-1: Monocyte chemoattractant protein – 1
MMP-2: Matric metallopeptidase – 2
PAH: Pulmonary arterial hypertension
PECAM-1: Platelet and endothelial cell adhesion molecule – 1
PF4: Platelet factor 4
RV: Right ventricle
RVSP: Right ventricular systolic pressure
TIMP-1: Tissue inhibitor of metalloproteinase – 1
TNF-α: Tumor necrosis factor alpha
